# Autism spectrum disorder in a boy with congenital insensitivity to pain with anhidrosis: a case report

**DOI:** 10.1186/s12887-022-03196-3

**Published:** 2022-03-11

**Authors:** Mi Zhang, Xueqin Cao, Ningbo Li, Guangyou Duan, Xianwei Zhang

**Affiliations:** 1 Department of Anesthesiology, Tongji Hospital, Tongji Medical College, Huazhong University of Science and Technology, Wuhan, 430030 China; 2 Department of Anesthesiology, The Second Affiliated Hospital, Chongqing Medical University, Chongqing, 400010 China

**Keywords:** Congenital insensitivity to pain with anhidrosis, Autism spectrum disorder, Developmental delays, Treatment and education of autistic and communication handicapped children and adults, Case report

## Abstract

**Background:**

In this case report, we described the past history, clinical manifestations, genetic characteristics and cognitive evaluation of a boy with congenital insensitivity to pain with anhidrosis (CIPA) who developed autism spectrum disorder (ASD).

**Case presentation:**

The boy had an early onset of CIPA at the age of 48 months, and was later diagnosed with ASD at 5 years old. Developmental delays in communication, social skills and the presence of maladaptive behaviors were observed in the patient. Professional treatments significantly improved the developmental delays.

**Conclusions:**

This case demonstrated that ASD may develop in children with CIPA, and pediatricians should be aware that if they suspect or identify a child with CIPA that they should also be screened for ASD using similar examination and diagnostic tools as shown in the present report. Moreover, therapeutic interventions for ASD was helpful for the remission of both diseases.

## Background

Congenital insensitivity to pain with anhidrosis (CIPA), also referred to as hereditary sensory and autonomic neuropathy type IV, is a rare inherited autosomal recessive disease. The incidence of this disorder is about 1 in 125 million, with few cases reported worldwide to date [1]. Our team has been monitoring cases of this disease for decades, and we have collected data on about 50 Chinese CIPA patients to date [2, 3]. Biallelic mutations in the neurotrophic tyrosine receptor kinase 1 gene (*NTRK1*, OMIM *191315) encoding tropomyosin-related kinase A (TrkA) results in this disease. TrkA is a receptor tyrosine kinase for nerve growth factor (NGF), and NGF promotes neurite outgrowth and maintains the survival of peripheral sensory and sympathetic postganglionic neurons originated from the neural crest, as well as central cholinergic neurons from the basal forebrain [4]. Therefore, the absence of sympathetic postganglionic neurons and primary afferent neurons with unmyelinated C-fibers contributes to the lack of the presence of anhidrosis and pain sensation in CIPA, respectively [4]. Lack of nerves that supply sweat glands can lead to recurrent episodic hyperthermia, while insensitivity to pain leads to joint injuries, with or without recurrent fractures, hip dislocations, and self-mutilating behavior. Moreover, many children with CIPA exhibit symptoms of varying degrees of developmental retardation and severe attention-deficit-hyperactivity disorder (ADHD) [5, 6].

Autism spectrum disorder (ASD) is a heterogeneous neurodevelopmental disorder, which is mainly characterized by abnormal language and social skills, as well as restricted behavioral patterns. The global prevalence of this disease is less than 1%, but higher in high-income countries [7]. Individuals with ASD all have very different clinical manifestations, but the principal features of the disorder are not influenced by culture, race, ethnicity, or socioeconomic status. In addition, ASD is partially accompanied by other diseases, including psychiatric and genetic disorders such as ADHD and fragile X syndrome [8]. Herein, we reported for the first time, the clinical, social, cognitive and psychiatric characteristics of a boy with CIPA who developed ASD.

## Case presentation

**Fig. 1:**
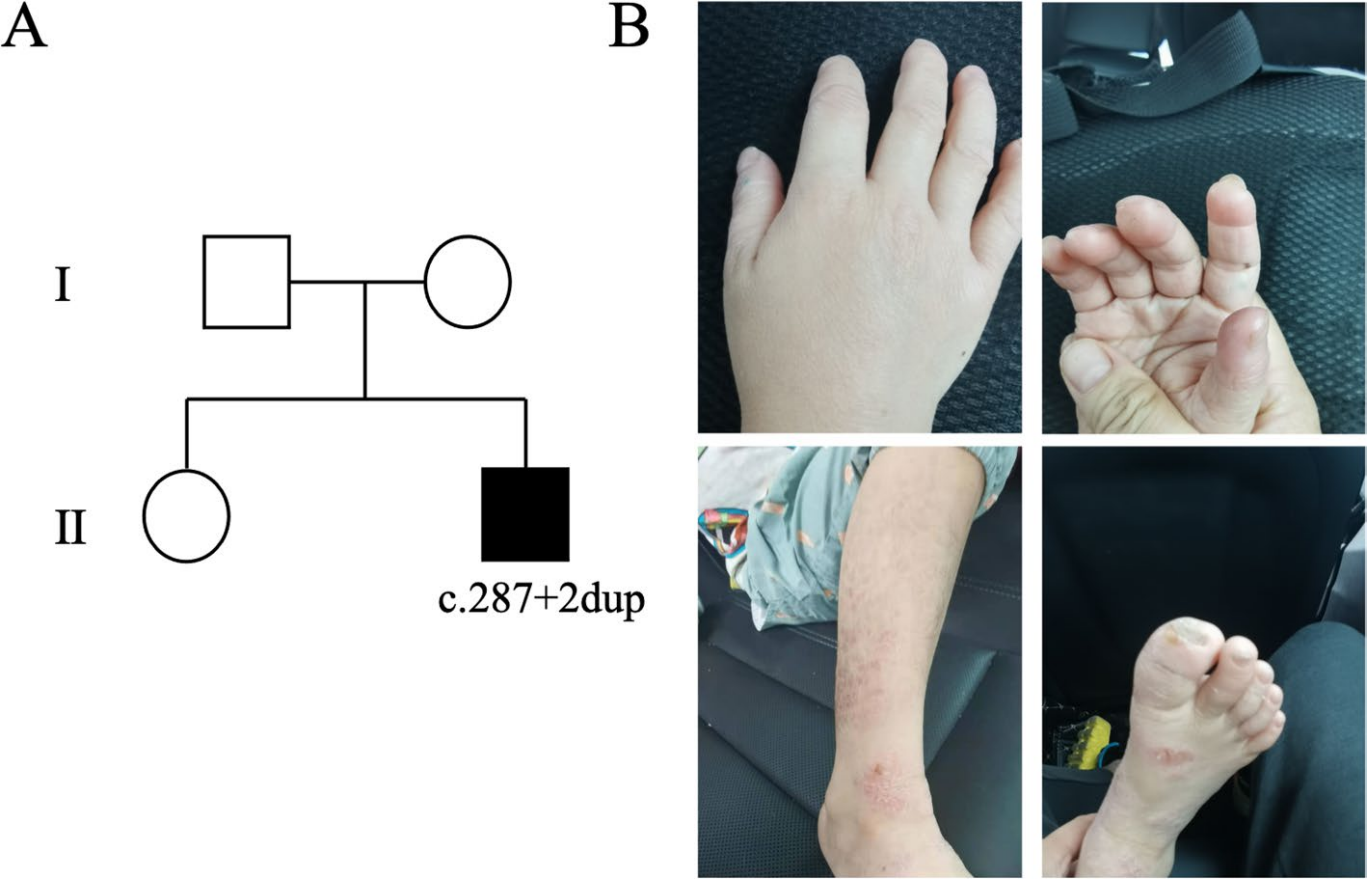
Phenotypic information of the patient. **A** Pedigree of the family. **B** Photographs showing certain clinical features of the patient

The 5-year-old Chinese boy was the second child of non-consanguineous parents and diagnosed with CIPA after a genetic test at the age of 48 months. Whole genome sequencing was performed to find his mutant gene, and a homozygous c.287 + 2dup mutation of *NTRK1* gene was identified, which was inherited from his father and mother, respectively (Fig. 1).

The boy developed high fever at 3 months of age. He suffered from recurrent episodes of uncontrolled fever due to unknown causes, and antipyretics were ineffective. In addition, he had no painful reaction to any injection. When the first teeth appeared, he began to bite his tongue, fingernails and toenails repeatedly without any feeling of pain. Subsequently, other members of his family realized that he was incapable of experiencing pain, and the self-mutilation continued until 48 months. When the boy started walking independently, he was more likely to fall down compared to his peers. However, he did not suffer from any bone fracture or dislocation. The patient could talk at 24 months, but could not speak complete sentences until 48 months. Notably, he often did not respond when other people called him. Hyperactivity and inattentiveness were also observed.

The boy was assessed by the pediatric and neurology departments, and physical examination showed dry skin, deformities in the fingers and toes, presentation of abdominal reflexes and loss of knee-jerk reflex. Behavioral observations indicated no response when others called his name, and also expression by simple language.

**Fig. 2:**
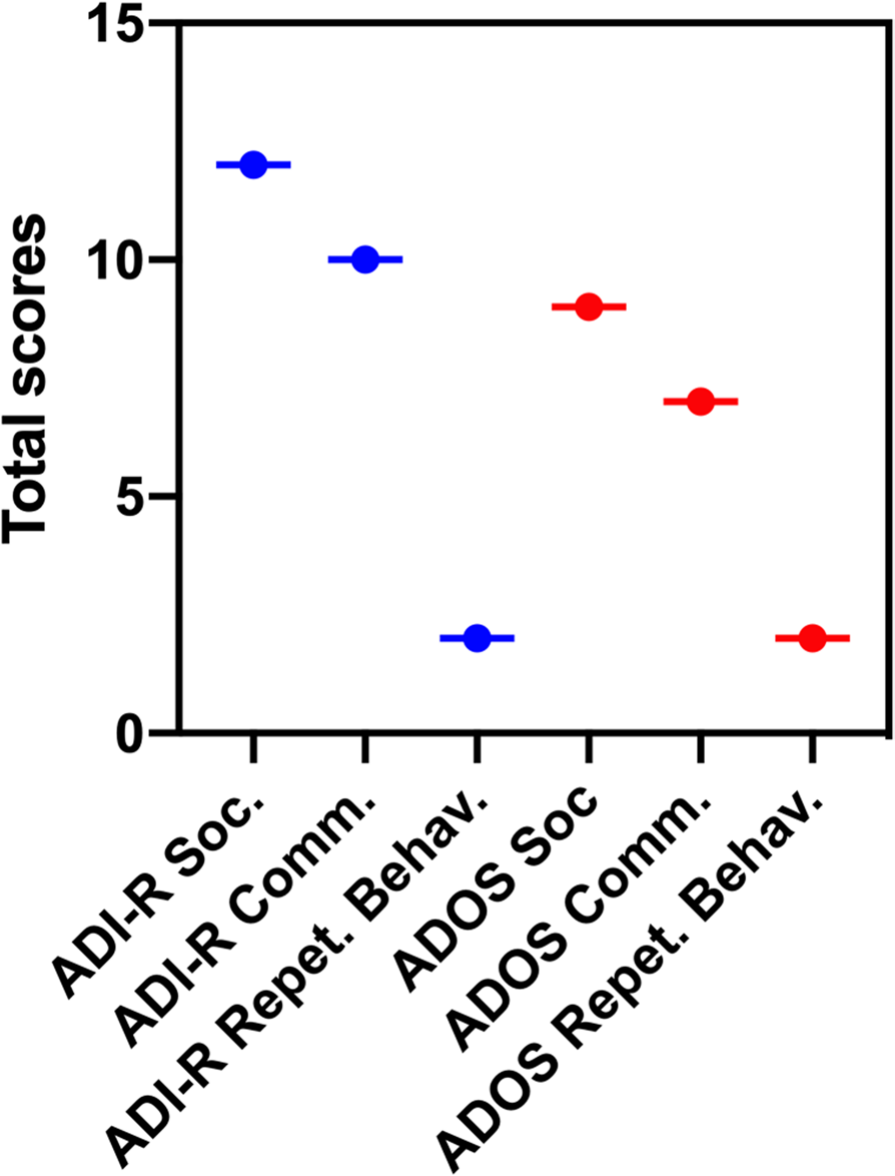
Total scores of the Autism Diagnostic Interview-Revised and the Autism Diagnostic Observation Schedule of the patient. This column chart represents total scores of ASD-related symptoms, which were assessed by ADI-R Social Impairment, ADI-R Communication Impairment, ADI-R Restricted and Repetitive Behavior, ADOS Social Impairment, ADOS Communication Impairment and ADOS Restricted and Repetitive Behavior. ASD, Autism Spectrum Disorder; ADI-R: Autism Diagnostic Interview-Revised; ADOS: Autism Diagnostic Observation Schedule

At the cognitive level, a composite score of the Full Scale IQ (FSIQ) of 67 was observed by an evaluation using the Wechsler Preschool and Primary Scale of Intelligence Fourth Edition (WPPSI-IV), which is an indicator of mild intellectual disability (Table 1). In addition, evaluation of the boy as per the Autism Diagnostic Interview-Revised (ADI-R) and the Autism Diagnostic Observation Schedule (ADOS) indicated that the scores of communication and social functioning exceeded the upper limit of diagnosis (Fig. 2). Furthermore, evaluation of developmental level of motor, communication skills and the presence of maladaptive behaviors based on the Psychoeducational Profile-Third Edition (PEP-3) indicated that the patient had a moderate developmental delay (Table 2). The patient was diagnosed as ASD based on these findings and clinical manifestations.

**Table 1:** Schedules scores of the Wechsler Preschool and Primary Scale of Intelligence Fourth Edition of the patient

Domain of Wechsler Preschool and Primary Scale of Intelligence Fourth Edition	Subtest of Wechsler Preschool and Primary Scale of Intelligence Fourth Edition	Scale score	Composite score
Verbal Comprehension Index	Similarities	3	69
	Information	4	
Visual Spatial Index	Block Design	4	67
	Object Assembly	4	
Fluid Reasoning Index	Matrix Reasoning	8	79
	Picture Concepts	5	
Working Memory Index	Picture Memory	4	67
	Zoo Location	4	
Processing Speed Index	Bug Search	4	61
	Cancellation	1	
Full Scale IQ		27	67

**Table 2:** Standard scores of Psychoeducational Profile-Third Edition and developmental age of the patient

Composites of Psychoeducational Profile-Third Edition	Domain of Psychoeducational Profile-Third Edition	Standard Scores	Percentile Ranks	Developmental Levels	Developmental Age (months)
Communication	Cognitive Verbal Preverbal	8	39	Moderate	33
	Expressive Language	11			44
	Receptive Language	11			41
Motor	Fine Motor	10	32	Moderate	42
	Gross Motor	8			25
	Visual Motor Limitation	12			40
Maladaptive Behavior	Affective Expression	12	64	Moderate	N
	Social Reciprocity	10			N
	Characteristic Motor Behavior	12			N
	Characteristic Verbal Behaviors	12			N

N: Not Applicable

After the diagnosis of ASD was established, the patient received intervention treatment of Treatment and Education of Autistic and Communication Handicapped Children and Adults (TEACCH), which was one of the evidence-based training models for people with ASD [9]. One-to-one coaching, exercise, feeding, game and music classes were carried out to improve his cognitive, communicate and social ability as well as his gross and fine motor functions. In addition, his parents were also trained for family training. After a range of treatments, language, motor and social skills of the boy were improved, which also had a significant effect on his main behavioral symptom of CIPA, such as self-mutilating.

## Discussion and conclusions

Patients with CIPA often have intellectual disability, in addition to personalities of hyperactivity, irritability and moodiness [2]. Levy Erez et al. implemented a formal evaluation of adaptive behavior and intelligence in 23 patients with CIPA and found a negative correlation between the intelligence quotient and age in patients [6]. Our team evaluated mental development of two children with CIPA on the basis of Gesell Developmental Schedules (GDS), which included adaption, motor, social functioning, and language functioning status [10]. The results indicated that the intellectual development of patients with CIPA gradually slowed down in the stages of early childhood development. In addition, researchers used the Wechsler Adult Intelligence Scale (WAIS) to assess adult patients with CIPA, and those patients generally had mild to moderate growth retardation [11, 12]. These data merely indicate that patients with different phenotypes of CIPA may experience profound developmental delay.

Developmental disorders of ASD are featured with social impairment, non-verbal and verbal communication difficulties, repetitive and stereotypical behavior, and restricted interests [9]. Approximately 40% of patients with ASD have developmental delays, and about 70% of them show some degree of intellectual disability [13]. The common denominator between CIPA and ASD is developmental delay, including behavior, emotion and intelligence. But for ASD, behavioral disorders, especially impaired social communication and interaction, are its main diagnostic criteria, these are not for CIPA. The presence of ASD in co-existing disorders can be identified through specialized assessment forms for ASD, such as ADOS and ADI-R. To date, this is the first report of a child with CIPA who developed ASD.

The causes of ASD include psychosocial, environmental and a multitude of genetic factors. The first evidence of genetic factors in ASD were found in patients with fragile X syndrome and tuberous sclerosis, and some of whom were accompanied by ASD. In addition, copy-number variants, which can be detected in patients with other developmental disorders, are also considered as risk variants for ASD. A few common variants, such as chromosome 16p11.2 deletions and duplications, and maternal 15q11-q13 duplications have been individually reported [8, 14]. In this report, in addition to the gene that caused CIPA, other mutations were also detected, such as *OPLAH* (c.2906G > A; c.1300G > A), *KCNV2* (c.66G > C; c.80G > A), *ABCC8* (c.3976G > A), *MYBPC3* (c.2543C > T), *PARN* (c.1637A > G; c.745C > T), *KARS1* (c.1467C > G). Of these, only *MYBPC3* had been reported in four patients with ASD [13]. However, only a few cases had been reported with this association, so whether ASD in this patient was induced by the mutations of these genes remains unknown, and more similar cases are needed to establish the correlation.

The boy was diagnosed with ASD in time, and the systematic treatments significantly improved his ASD-related symptoms, which also had a significant effect on his main behavioral symptom of CIPA. Most patients with CIPA have intellectual disability, emotional instability, hyperactivity and other characteristics. However, the parents and doctors may attribute these symptoms to CIPA, without considering other diseases. So delayed diagnosis and under-treatment for ASD are possible. We propose that coexisting conditions in more cases should be professionally evaluated, and early intervention of typical symptoms and developing an appropriate training system at an early age might improve the outcome.

In summary, this case demonstrated that ASD may develop in children with CIPA, and pediatricians should be aware that if they suspect or identify a child with CIPA that they should also be screened for ASD using similar examination and diagnostic tools as shown in the present report. We propose that the therapeutic approaches used for alleviating the developmental delay in CIPA children with ASD may be similarly effective in children without ASD.

## Data Availability

All data generated or analysed during this study are included in this published article.

## Abbreviations

CIPA: Congenital insensitivity to pain with anhidrosis
ASD: Autism spectrum disorder
NTRK1: Neurotrophic tyrosine receptor kinase 1
TrkA: Tropomyosin-related kinase A
NGF: Nerve growth factor
ADHD: Attention-deficit-hyperactivity disorder
FSIQ: Full scale IQ
WPPSI-IV: Wechsler preschool and primary scale of intelligence fourth edition
ADI-R: Autism diagnostic interview-revised
ADOS: Autism diagnostic observation schedule
PEP-3: Psychoeducational profile-third edition
TEACCH: Treatment and education of autistic and communication handicapped children and adults
GDS: Gesell developmental schedules
WAIS: Wechsler adult intelligence scale
